# IOERT versus external beam electrons for boost radiotherapy in stage I/II breast cancer: 10-year results of a phase III randomized study

**DOI:** 10.1186/s13058-021-01424-9

**Published:** 2021-04-13

**Authors:** Antonella Ciabattoni, Fabiana Gregucci, Gerd Fastner, Silvio Cavuto, Antonio Spera, Stefano Drago, Ingrid Ziegle, Maria Alessandra Mirri, Rita Consorti, Felix Sedlmayer

**Affiliations:** 1 Department of Radiotherapy, San Filippo Neri Hospital, ASL Roma 1, Rome, Italy; 2 Department of Radiation Oncology, Miulli General Regional Hospital, Acquaviva delle Fonti, Bari, Italy; 3 Department of Radiotherapy and Radio-Oncology, Paracelsus Medical University Hospital Salzburg, Landeskrankenhaus, Salzburg, Austria; 4 Infrastructure Research and Statistics, Clinical Trials and Statistics Unit, AUSL-IRCCS, Reggio Emilia, Italy; 5 Department of Radiotherapy, San Giovanni di Dio Hospital, ASP Agrigento, Agrigento, Italy; 6 Department of Breast and Reconstructive Surgery, Sando Pertini Hospital, Rome, Italy; 7 Medical Physics Unit, San Filippo Neri Hospital, ASL Roma 1, Rome, Italy

**Keywords:** Breast cancer, Intraoperative radiotherapy, Electrons, Tumor bed boost, Local control, Toxicity, Cosmesis

## Abstract

**Background:**

Intraoperative radiotherapy with electrons (IOERT) boost could be not inferior to external beam radiotherapy (EBRT) boost in terms of local control and tissue tolerance. The aim of the study is to present the long-term follow-up results on local control, esthetic evaluation, and toxicity of a prospective study on early-stage breast cancer patients treated with breast-conserving surgery with an IOERT boost of 10 Gy (experimental group) versus 5 × 2 Gy EBRT boost (standard arm). Both arms received whole-breast irradiation (WBI) with 50 Gy (2 Gy single dose).

**Methods:**

A single-institution phase III randomized study to compare IOERT versus EBRT boost in early-stage breast cancer was conducted as a non-inferiority trial. Primary endpoints were the evaluation of in-breast true recurrences (IBTR) and out-field local recurrences (LR) as well as toxicity and cosmetic results. Secondary endpoints were overall survival (OS), disease-free survival (DFS), and patient’s grade of satisfaction with cosmetic outcomes.

**Results:**

Between 1999 and 2004, 245 patients were randomized: 133 for IOERT and 112 for EBRT. The median follow-up was 12 years (range 10–16 years). The cumulative risk of IBTR at 5–10 years was 0.8% and 4.3% after IOERT, compared to 4.2% and 5.3% after EBRT boost (*p* = 0.709). The cumulative risk of out-field LR at 5–10 years was 4.7% and 7.9% for IOERT versus 5.2% and 10.3% for EBRT (*p* = 0.762). All of the IOERT arm recurrences were observed at > 100 months’ follow-up, whereas the mean time to recurrence in the EBRT group was earlier (55.2 months) (*p* < 0.05). No late complications associated with IOERT were observed. The overall cosmetic results were scored as good or excellent in physician and patient evaluations for both IOERT and EBRT. There were significantly better scores for IOERT at all time points in physician and patient evaluations with the greatest difference at the end of EBRT (*p* = 0.006 objective and *p* = 0.0004 subjective) and most narrow difference at 12 months after the end of EBRT (*p* = 0.08 objective and *p* = 0.04 subjective analysis).

**Conclusion:**

A 10-Gy IOERT boost during breast-conserving surgery provides high local control rates without significant morbidity. Although not significantly superior to external beam boosts, the median time to local recurrences after IOERT is prolonged by more than 4 years.

## Introduction

Currently, the standard local treatment for patients with early-stage breast cancer includes lumpectomy, sentinel lymph node biopsy (SLB), and whole-breast irradiation (WBI). WBI is delivered as external beam RT (EBRT) with a total biologically equivalent dose (BED) around 50 Gy in conventional or nowadays increasingly hypofractionated schedules. In patients deemed at higher risk for local recurrence, WBI is followed by a boost to the tumor bed, mostly by 10–16 Gy in 5–8 daily fractions [1, 2]. Boost methods comprise either EBRT, brachytherapy (BT), or intraoperative radiotherapy (IORT) [3].

IORT is performed with different energies, from kV-based systems up to Linac-based electrons (IOERT), nowadays mostly delivered with mobile linear accelerators in order to avoid patient transportation, thus reducing perioperative infections [4]. Among the various boost techniques, IORT is attractive for several reasons: the avoidance of “spatial” [3] as well as “temporal” missings [5], since the dose is delivered at utmost precision to a given tumor bed during surgery, and tumor cell repopulation between surgery and adjuvant RT is reduced or prevented. In addition, treatment volumes for IOERT are smaller than for EBRT, the dose fall-off to surrounding normal tissues is very steep and the skin as an organ at risk for toxicity is not irradiated. These factors should contribute to better tissue tolerance [6]. Finally, when compared to EBRT boosts, IORT saves the need for an additional week of daily treatment sessions.

These considerations prompted a randomized prospective study on early-stage breast cancer patients hypothesizing that in terms of local control, an IOERT boost of 10 Gy prior to a 50-Gy WBI is non-inferior to a fractionated 10-Gy EBRT boost after WBI, while obtaining a low toxicity profile and good esthetical result. The aim of the present paper is the long-term evaluation of local control, toxicity, and cosmetic outcomes.

## Material and methods

A randomized phase III monocentric study on IOERT versus an EBRT boost in early breast cancer patients was performed from April 1999 to April 2004 as a non-inferiority trial. Primary endpoints were the evaluation of in-breast true recurrence (IBTR) and out-field local recurrence (LR) rates, the incidence of acute and late toxicities, and cosmetic results. Secondary endpoints were overall survival (OS) and disease-free survival (DFS). IBRT was defined as the reappearance of the same histologic tumor within 3 cm from the former primary lesion, while the out-field LR was defined as any elsewhere recurrence within the irradiated breast. The toxicity evaluation was assessed using the European Organization for Research and Treatment of Cancer (EORTC) scale, at the end of WBI and during the follow-up as acute (before 3 months) and late (after 3 months). The cosmetic result was objectively evaluated by the same physician, based on five parameters (hyperpigmentation, telangiectasias, hypertrophic scar, profile asymmetry, and difference in consistency), and scored according to the Harvard Scale [7]. This scoring compares an overall cosmetic impression of the treated breast with the untreated one, categorizing the results as excellent, good, fair, or poor. The same cosmetic outcomes were reported by patients themselves with a self-assessed questionnaire based on the Harvard Scale and compared with the physician’s judgment. The first assessment of the cosmetic result was done before starting WBI and at the end of it. The same evaluation approach was performed at three time points after the end of RT, at 1 and 6 months and 1 year thereafter. At this time, the evaluation of the cosmetic result was terminated, considering these data as representative and not subject to subsequent major changes.

OS was calculated from the date of WBI to the death for any cause or last follow-up date. DFS was defined as any event of local and distant disease recurrence and calculated from the date of WBI to the relapse or last follow-up date.

### Patients selection and clinical records

Patients’ selection was based on the following inclusion criteria: age 18–75 years, female sex, histology- or cytology-proven invasive breast carcinoma, clinically staged cT1–cT2 cN0–N1 without evidence of distant metastases (M0), ECOG performance status < 2, and no previous breast radiotherapy. Staging was done according to the American Joint Committee on Cancer (AJCC fifth edition, 1997). The exclusion criteria were documented multicentricity or multifocality disease, in situ ductal (DCIS) or lobular carcinoma (LCIS) histology without invasive component, Paget disease, extended intraductal component (EIC), distant metastases, pregnancy, breastfeeding, and inability to give an informed consent. Pre-treatment work-up included mammography, breast and axillary ultrasound, tumor biopsy, staging exams (chest X-ray, bone scan, liver sonography), and lab evaluation of menopausal status.

### Surgery

All patients underwent quadrantectomy or wide local excision of the primary, with a free margin of at least 10 mm for invasive and 5 mm for in-situ disease, according to the standards of time at study initiation. Margins were intraoperatively verified by fast-frozen sections. If the final histopathology revealed less, a re-resection was recommended and in many cases extended to the pectoral fascia. In all patients, surgical clips were positioned to identify the tumor bed. Standard SLB was performed, in case of positive nodes followed by axillary dissection. The specimen was examined with X-ray in the operating theater to ensure complete excision of the lesion and to help with the assessment of the adequacy of the margins. In case of close margins for microcalcification, further tissue was excised.

### Radiation treatment

IOERT was performed with a mobile linear accelerator Novac7 (Hitesys SPA, SIT). A single dose of 10 Gy was prescribed to the 90% reference isodose, covering the planning target volume (PTV) which included the former tumor volume with a radial margin of 2 cm. Target depths ranged between 1.4 and 1.9 cm, electron energies between 7 and 9 MeV, tube sizes between 40 and 80 mm in diameter, and with 0–15° beveled applicators. Surgical clips were positioned at the edge of the irradiated areas. To minimize the radiation-induced side effects at the applicator surface, a distance of more than 5 mm between the skin and applicator was recommended. All patients received conventional 50 Gy WBI in 2 Gy per fraction, with 6 to 10 MV photons and opposed tangential field technique after 3D conformal planning. WBI included irradiation of the regional lymph nodes with 50 Gy in ≥ pN2 situations. In patients not undergoing systemic chemotherapy, a delay of at least 5 weeks was required between IOERT and WBI start. In the EBRT boost arm, a dose of 10 Gy in 5 fractions to the 90% reference isodose was delivered with a single 6–12-MeV electron beam portal to the clip-marked tumor bed.

### In vivo dosimetry

In order to check the agreement between dose prescription and dose delivered with IOERT, in vivo “on-line” dosimetry using metal oxide silicon field effect transistors (MOSFETs) was performed from 2002 in a total of 20 patients in the IOERT boost arm. The MOSFET, inserted in a sterile plastic wrap, was placed on the treatment surface.

### Adjuvant treatment

Adjuvant systemic treatment was performed according to the international guidelines of that time, mostly CMF-based. A delay of around 30 days was required between chemotherapy and WBI. Concomitant WBI and hormonal therapy were allowed.

### Follow-up

Follow-up visits were scheduled after 1, 6, and 12 months after radiotherapy and annually thereafter. The cosmetic result was objectively evaluated by a physician and patients according to the Harvard Scale, and the results were categorized as excellent, good, fair, or poor. The cosmetic evaluation was performed at five time points: before RT, end of RT, 1 month, 6 months, and 1 year after RT. For oncologic endpoints, follow-up was continued annually comprising clinical exams, mammography, abdominal ultrasound, lab test, and chest X-ray.

### Statistical methods

The study was designed as a non-inferiority randomized trial. Considering a power of 80% and an alpha error of 0.05, the R statistical software was used applying the one-side method for two proportions, resulting in a required sample size of 316 patients per study arm. A scheme of simple randomization (AB-AB) that provided two arms of patients with similar clinical characteristics was chosen. A statistical descriptive analysis was performed to summarize the principal clinical, disease, treatment, and outcome characteristics between the two treatment groups. The risks of IBTR and out-field LR were performed by the Kaplan-Meier method, and the evaluation of the difference between IOERT boost arm versus EBRT boost arm was calculated by the log-rank test, also for OS and DFS. For cosmetic results, the chi-square test was applied to compare the different proportions for each group. *p* value ≤0.05 was considered as statistically significant. Data were examined in June 2019.

## Results

**Fig. 1:**
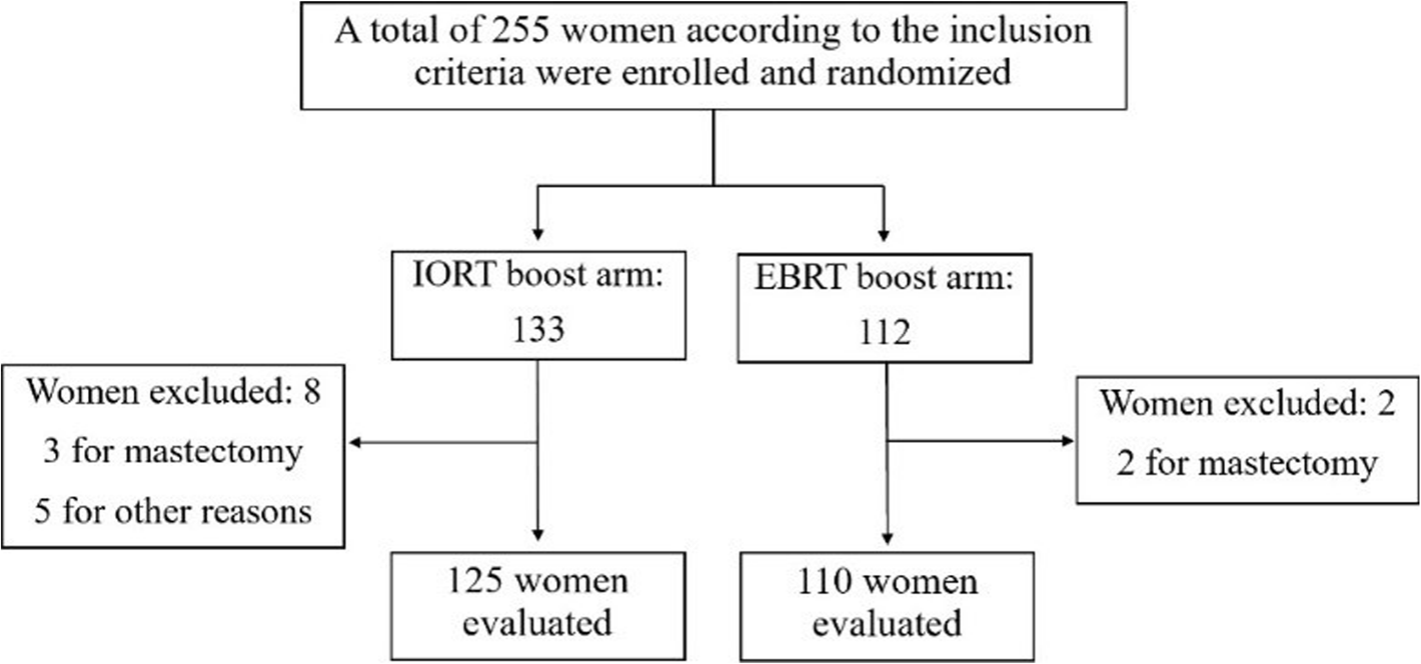
Recruitment and randomization

**Fig. 2:**
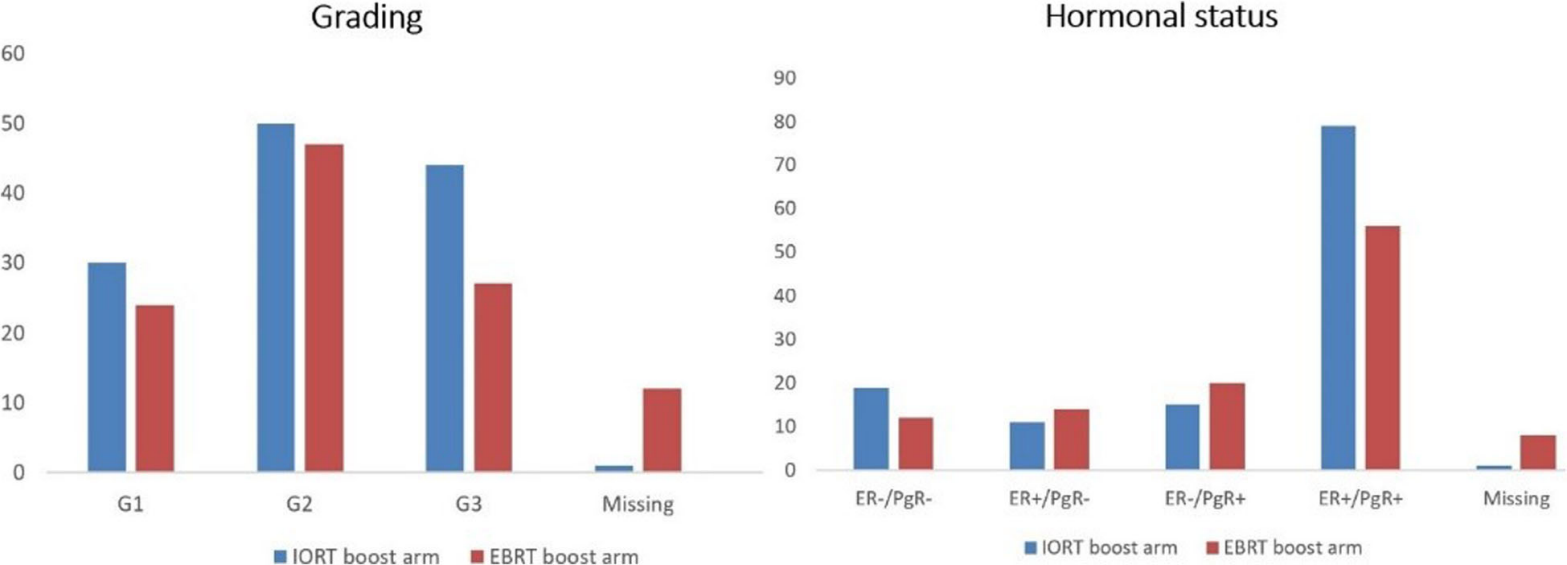
Distribution of absolute frequencies by grading and hormonal status

From April 1999 to April 2004, a total of 245 women were enrolled and randomized in the study. The accrual goal was not achieved due to the expiry of the insurance policy provided by the protocol and the unavailability of funds for its renewal. Of the 245 patients, 10 were excluded from this analysis because five underwent mastectomy for multi-centricity, multi-focality, or positive margins. The other five were considered out of protocol for different reasons: 2 underwent radiotherapy in other hospitals, 2 were not evaluable for missing one or more histological data, and one patient refused external beam treatment. Excluding these, overall, 235 patients were evaluated: 125 in the IOERT boost arm and 110 in the EBRT boost arm, as shown in Fig. 1. All women were assessed for pathologic tumor size, nodal status, surgical margins, histology, grading, expression of hormone receptors, and systemic chemotherapy. The main characteristics are listed in Table 1, and the comparison of biological features between the two groups is represented in Fig. 2. Although the sample size in the IOERT boost group was slightly bigger than in the EBRT boost group, patients and tumor characteristics were almost equally balanced except for G3 gradings, with 44/125 cases (35%) in the IOERT arm versus 27/110 cases (24%) in the EBRT group.

**Table 1:** Patients, tumor, and systemic therapy characteristics

	IOERT boost arm	EBRT boost arm	*p* value
Women in each arm	125 (51%)	110 (49%)	
Median age (range)	56.3 years (29–75)	56.2 years (34–75)	0.96**
Menopausal status			0.81*
Pre	37 (30%)	31 (28%)	
Post	88 (70%)	79 (72%)	
Disease laterality			0.73*
Right breast	54 (43%)	50 (45%)	
Left breast	71 (57%)	60 (55%)	
Histology			0.46*
IDC	61 (49%)	59 (54%)	
ILC	17 (14%)	11 (10%)	
DCIS	3 (1.4%)	3 (2.6%)	
IDC+DCIS	32 (26%)	24 (22%)	
Medullary	6 (5%)	4 (3.5%)	
ILC+ILCS	5 (4%)	5 (4.4%)	
Missing	1 (0.6%)	4 (3.5%)	
Pathologic T stage			0.38*
pT1	96 (77%)	79 (72%)	
pT2	28 (22%)	22 (20%)	
pT3–pT4	1 (1%)	1 (1%)	
Missing	0	8 (7%)	
Pathologic N stage			0.55*
pN0	82 (65%)	68 (62%)	
pN1	38 (30%)	32 (29%)	
pN2	2 (2.1%)	1 (1%)	
pN3	1 (0.8%)	1 (1%)	
Missing	2 (2.1%)	8 (7%)	
Adjuvant chemotherapy			0.61*
Yes	37 (30%)	36 (33%)	
Not	85 (68%)	64 (58%)	
Missing	3 (2%)	10 (9%)	
Hormone therapy			0.66*
Yes	105 (84%)	90 (81%)	
Not	19 (15%)	12 (11%)	
Missing	1 (1%)	8 (8%)	

*Abbreviations*: *IDC*, invasive ductal carcinoma; *ILC*, invasive lobular carcinoma; *DCIS*, ductal carcinoma in situ

**Mann-Whitney test

*Chi-square test

All 235 patients underwent lumpectomy. In 230 patients, negative margins were achieved at the first operation, whereas 5 patients (2 in the IOERT arm and 3 in the EBRT arm) underwent re-excision for positive margins. Women with close margins (less than 1 cm) were included in the analysis. All patients received WBI with a total dose of 50 Gy in 5 weeks. Adjuvant chemotherapy was administered in 37 patients (30%) in the IOERT boost arm and 36 patients (33%) in the EBRT group arm, while hormonal therapy was given in most cases, 84% and 81% in IOERT and EBRT group, respectively.

### Local failure and survival

**Fig. 3:**
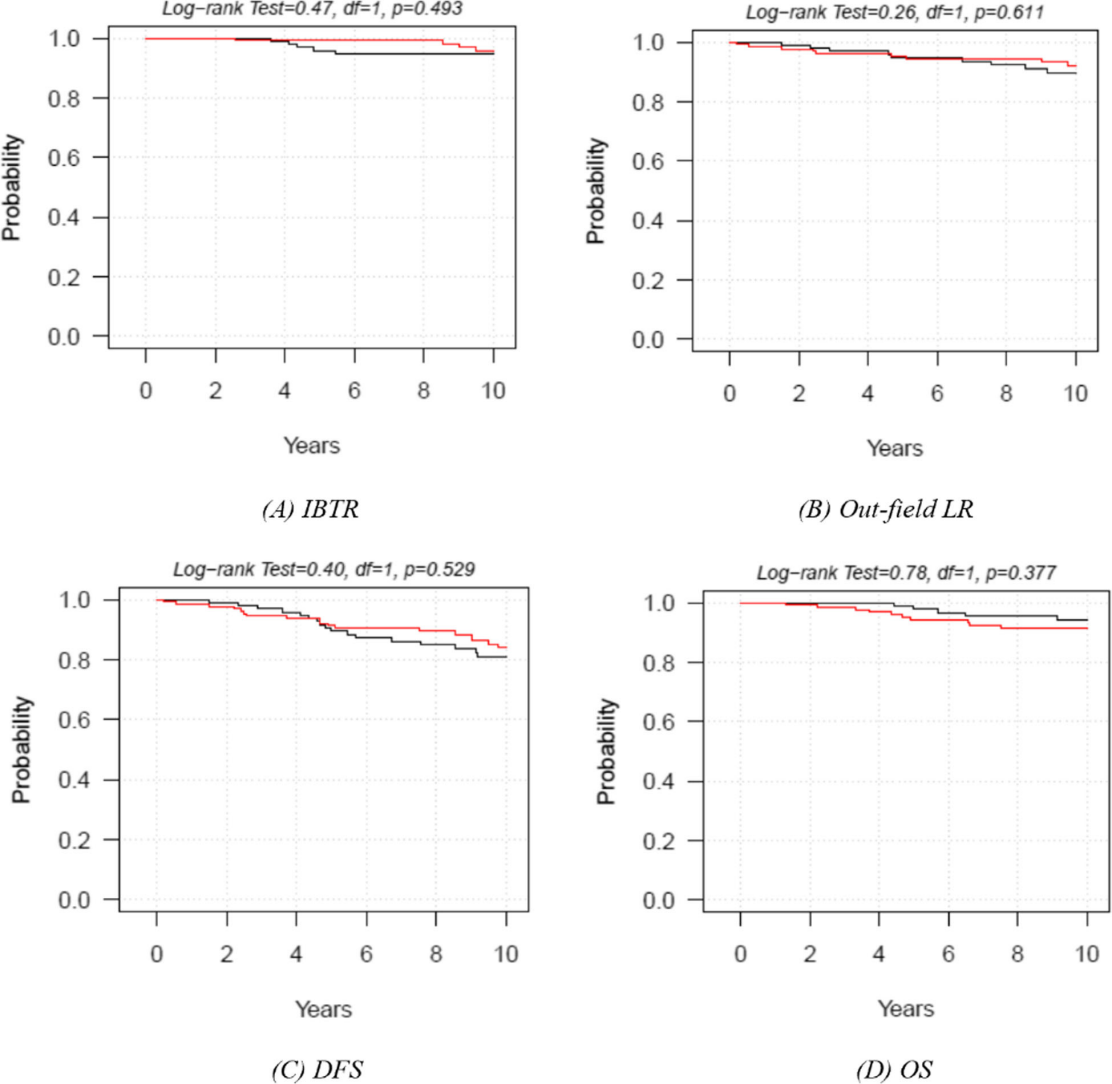
Kaplan-Meier curves of IBTR (**a**), out-field LR (**b**), DFS (**c**), and OS (**d**). IBTR, in breast true recurrence; LR, local recurrence; DFS, disease-free survival; OS, overall survival. IEORT arm (red line); EBRT arm (black line)

With a median follow-up of 12 years (range 10–16 years), altogether, 38 in-breast recurrences were noted. In the IOERT boost group, 19 local recurrences (15.2%) were observed: 4 of them (3.2%) were rated as IBTR and the others as anywhere else (marginal or in a different quadrant). In the EBRT boost group, 19 local recurrences (17.3%) were detected: 5 of them (4.5%) were classified as IBTR, and the others were in a different quadrant (Table 2). Of note, all of the IOERT arm recurrences were observed at more than 100 months follow-up, whereas the mean time to recurrence in the EBRT group was much earlier (55.2 months). The cumulative risk of IBTR at 5 and 10 years was 0.8% and 4.3% in the IOERT group and 4.2% and 5.3% in the EBRT group (*p* = 0.493), respectively (Fig. 3a). The cumulative risk of out-field LR at 5 and 10 years amounted to 4.7% and 7.9% in the IOERT group and 5.2% and 10.3% in the EBRT group (*p* = 0.611), respectively (Fig. 3b).

**Table 2:** In-breast true recurrence (IBTR) and out-field local recurrence (LR) at 5 and 10 years

Treatment	IBTR number at 5 years	Risk of recurrence at 5 years	IBTR number at 10 years	Risk of recurrence at 10 years	Log-rank test *p* value
**IOERT**	1	**0.8%**	3	4.3%	0.493
**EBRT**	4	**4.2%**	1	5.3%	
Treatment	Out-field LR number at 5 years	Risk of recurrence at 5 years	Out-field LR number at 10 years	Risk of recurrence at 10 years	Log-rank test *p* value
**IOERT**	6	4.7%	9	7.9%	0.611
**EBRT**	5	5.2%	9	10.3%

In the IOERT arm, 3 patients developed distant metastases (brain and bone), while in the EBRT arm, 7 patients showed distant relapses (bone, liver, and lung). After IOERT, the DFS rates at 5 and 10 years were 91.4% (IC95% 84.9–95.1%) and 84% (IC95% 75.7–89.7%), respectively, while in the EBRT arm, the DFS rates at 5 and 10 years amounted to 90.6% (IC95% 82.7–95%) and 80.9% (IC95% 70.9–87.7%), respectively (log-rank test *p* value = 0.529) (Fig. 3c). As to the entire study population, 21 patients died. After IOERT, the OS rates at 5 and 10 years were 94.5% (IC95% 88.7–97.3%) and 91.6% (IC95% 84.9–95.4%), while OS for the EBRT groups at 5 and 10 years amounted to 99% (IC95% 92.8–99.9%) and 94.3% (IC95% 86.9–97.6%), respectively (log-rank test *p* value =0.377) (Fig. 3d).

### Toxicity

As acute toxicity, 12 patients developed post-surgical seromas (7 in the IOERT arm, 5 in the EBRT group) and 7 wound healing problems occurred (7.8%), 3 of them in the IOERT arm. Late reactions associated with IOERT were not observed, except two cases of liponecrosis in the treatment area 2 and 3 years after surgery. These were mammographic findings only without any subjective or cosmetic impairment. These two patients underwent a second surgery that confirmed the benign nature.

### Cosmesis

**Fig. 4:**
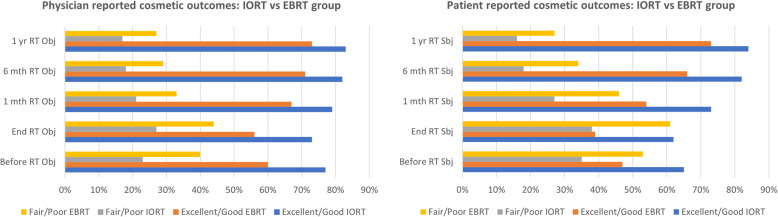
Cosmetic outcome in the IOERT and EBRT group according to Harvard Scale over the time points

The overall cosmetic results were rated as excellent/good in the majority of cases, as reported by physicians and patients (Fig. 4). Cosmetic outcomes were significantly better in the IOERT group compared to the EBRT group, and the difference remained significant at any examination, both in the physician’s evaluation and in patients’ evaluation (Table 3).

**Table 3:** Cosmetic outcomes according to Harvard Scale by (A) physician and (B) patients

Time point	Cosmetic rating	IOERT (125)	EBRT (110)	*p* value*
(A) Physician’s evaluation				
Before RT	Excellent/good	96 (77%)	66 (60%)	0.01
	Fair/Poor	29 (23%)	44 (40%)	
End RT	Excellent/good	91 (73%)	62 (56%)	0.006
	Fair/poor	34 (27%)	48 (44%)	
1 month after RT	Excellent/good	99 (79%)	74 (67%)	0.03
	Fair/poor	26 (21%)	36 (33%)	
6 months after RT	Excellent/good	103 (82%)	78 (71%)	0.05
	Fair/poor	22 (18%)	32 (29%)	
12 months after RT	Excellent/good	104 (83%)	80 (73%)	0.08
	Fair/poor	21 (17%)	30 (27%)	
(B) Patients’ evaluation				
Before RT	Excellent/good	81 (65%)	52 (47%)	0.006
	Fair/poor	44 (35%)	58 (53%)	
End RT	Excellent/good	78 (62%)	43 (39%)	0.0004
	Fair/poor	47 (38%)	67 (61%)	
1 month after RT	Excellent/good	91 (73%)	59 (54%)	0.001
	Fair/poor	34 (27%)	51 (46%)	
6 months after RT	Excellent/good	103 (82%)	73 (66%)	0.005
	Fair/poor	22 (18%)	37 (34%)	
12 months after RT	Excellent/good	105 (84%)	80 (73%)	0.04
	Fair/poor	20 (16%)	30 (27%)	

*Chi-square test

## Discussion

Since 1990, quadrantectomy plus WBI was the standard of care for early-stage breast cancer [8]. Over the years, the by far most widely used dose schedule was around 50 Gy in daily fractional sizes of 1.8–2 Gy. The majority of institutions added an extra dose to the tumor bed, mostly by electrons or brachytherapy. Since approximately 85% of (at least first) in-breast recurrences are confined to the same quadrant of the primary lesion [9], it appears reasonable to counteract by delivering a higher dose of radiation to this area.

This fact gave rise to partial breast irradiation (PBI) strategies where treatment is reduced just to this area [10]. Among the techniques investigated in large prospective randomized trials were BT [11, 12], EBRT [13–15], and IORT [16, 17]. For well-selected patients with a (very) low-risk breast cancer, PBI with interstitial brachytherapy, EBRT, or IOERT is nowadays considered a viable alternative to WBI also outside clinical trials [18].

EORTC 22881-10882 is the most important trial that investigated the role of a dose augmentation in the tumor bed; 5569 women were randomized to WBI (50 Gy) or WBI plus 16 Gy boost [10]. With a median follow-up of 5.1 years, the results showed the clear efficacy of the additional dose in terms of local control, independent from adjuvant systemic therapy, and the benefit was more evident for patients 40 years old or younger. In an update with 20 years follow-up, it was confirmed that a boost improves local control, at the cost of a higher risk of developing moderate fibrosis [19].

More recently, higher tumor bed doses have been reported to be detrimental for esthetic results: in the *Young Boost Trial* by Brouwers et al. [20], a randomization of 16 Gy versus 26 Gy boost dose was made on patients > 50 years of age, using external photons (73% versus 74%), electrons (22% versus 18%) or interstitial brachytherapy (1%). Cosmetic results were significantly worse in high-dose patients, with a significant correlation between the grade of fibrosis and the cosmetic outcomes.

Identification and treatment of a clinical target volume (CTV) on the basis of the surgical scar might lead to important underdosages with subsequent implications for local control [21]. Computer tomography images could help in the localization of the area to treat, with the caveat of a large significant interobserver variability [22] especially when a seroma is not clearly visible. Furthermore, the volume of the excision site tends to change during the course of WBI [23], adding an uncertainty that can be solved only by increasing margins of CTV.

By using IOERT as a boost, issues related to the correct identification of the target are resolved by direct visualization of the area to treat, minimizing the possibility of a geographical miss. Furthermore, the results of a pilot study conducted on 50 women treated with IOERT boost (9–20 Gy) followed by WBI (50 Gy, 2 Gy/Fr) showed good to excellent cosmetic score in all patients examined with a median follow-up of 9.1 years [4].

Assuming an alpha/beta of 4 for breast tumor, one fraction of 10 Gy corresponds to an EQD2 of about 23.3 Gy. Unlike in the Brouwers et al.’s study [20], a delivery of this dose equivalent by IOERT does not compromise the cosmetic result.

Regarding the use of IOERT as a boost, there is solid retrospective experience published [24–26]. The largest pooled analysis was promoted by the International Society of Intraoperative Radiation Therapy (ISIORT) comprising 1109 unselected patients from 7 different centers using the same IOERT and WBI doses: 10 Gy IOERT as a boost and 50–54 Gy (1.7–2.0 Gy/Fr) WBI. At a median follow-up of 72.4 months, 16 in-breast recurrence events occurred and a tumor control rate of 99.2% was achieved [25].

In the recently published data by the Salzburg group [26], a cohort of 770 breast cancer patients forming a subgroup of the ISIORT pooled analyses was re-analyzed after a longer follow-up, followed for 10 years, analyzed in terms of local control (LC) and survival outcome. After a median follow-up of 121 months, local control (LC) still amounted to 97.2%. In a multivariate analysis, HER2-positive and triple-negative breast cancer subtype (TN) turned out to be significant negative predictors for IBRs, but no longer high tumor grade (G3) or a positive nodal status, which was in contrast to previous findings.

A prospective phase II trial by Ivaldi et al. [27] explored the effectiveness of IOERT as a boost (12 Gy) combined with hypofractionated whole-breast radiotherapy (2.85 Gy in 13 daily fractions) in 204 patients, with radiation toxicity as the primary end point. Acute skin G0–G2 reactions occurred in 96.2% (97.8% in the boost area) and G3 in 3.8% (2.2% of which in the boost area) at the end of treatment.

In the HIOB protocol [28], a prospective multicenter single-arm trial (NCT01343459), an IOERT boost (11.1 Gy) followed by moderately hypofractionated WBI (40.5 Gy/15 fractions), is investigated for toxicity and oncologic outcome. For the first 583 patients, treatment toxicity was reported after a median follow-up of 45 months, revealing excellent tissue tolerance of this regimen: CTCAE score 0/1 acute effects were noted in 91% (end of treatment) and LENT-SOMA 0/1 late effects in 96.5% (91–100) at 6 years.

To our knowledge, the present study is the only randomized study that compares a boost with IOERT with a classic EBRT approach. Our analysis revealed that the average time to recurrence in IOERT arm patients was markedly longer than in the control arm. At 5 years FU, local control was obviously in favor of IOERT with 0.8% LRR versus 4.2% following EBRT (*p* 0.001). At 10 years FU, this advantage reduces, maintaining a slightly better performance for IOERT (4.3% in IOERT boost arm versus 5.3% in the standard group [n.s.]). Both loco-regional control and overall survival showed no significant differences between the two treatment arms, however, again with a trend in favor of IOERT.

The current rates of DFS and OS at 5 years compared to the modern treatment approaches for breast cancer are stably low and even better in the last years both for the improvement of the techniques and for greater radiobiological knowledge. Both of these improvements allow us to use optimal treatment and fractionation approaches [29]. In particular, the use of concomitant boost represents an optimization of dose delivery without a lengthening of the treatment times. However, IOERT remains a potentially advantageous boost technique because, from a radiobiological point of view, it allows to administer more than a double equivalent dose to the tumor bed.

Limitations of this study were related to the lack of information on modern biological prognostic factors like Ki-67 and HER2 status, which is coherent with the period when the study was designed. For the same reason, chemotherapy was mainly based on CMF instead of anthracyclines and/or targeted therapies.

The most important limitation is the early interruption of the study and, thus, the limited accrual that can explain in terms of randomness a slight difference in sample size between the 2 study groups (125 patients in the IEORT group versus 110 patients in the EBRT group). Nonetheless, a major strength of the work is the very long follow-up for oncologic endpoints.

Cosmetic evaluation was stopped at 1 year FU, which might nowadays be considered as insufficient for the final assessment. However, in the HIOB study with a comparable RT regimen, there was no further notable cosmetic deterioration after 1 year of follow-up.

Another potential bias could be that cosmetic valuation before EBRT was better in the IOERT group with respect to the EBRT boost group. However, even if it is difficult to think of a better esthetic result linked to IOERT, it is not possible to assess if this difference was caused by major attention from the doctors and/or greater patient satisfaction for the treatment. The IOERT group already had better “starting conditions” before EBRT, and this can be considered a potential bias of the study.

To our mind, our results at least confirm the oncologic iso-efficacy of the IOERT boost versus the EBRT boost while obtaining better cosmetic results. The lack of significance between the two groups could well be caused by underpowering of the analysis. Another major finding is that an IOERT boost seems to postpone the relapse event decisively along the timeline. This effect is not limited to the true local recurrences but also observed for the out-quadrant relapses. Although not significant, it is interesting that especially at 10 years, the number of out-field recurrences is in favor of the IOERT group: 7.9% versus 10.3% of the standard group. Of note, G3 grading as an important risk factor for local recurrence [30] was more represented in the IOERT cohort. Moreover, in the long follow-up, no recurrences have occurred in young patients (under the age of 45 years), confirming that dose intensification has a major clinical significance in this group of patients.

## Conclusions

IOERT boost is an advantageous approach for breast cancer patients who need dose escalation to the tumor bed, reducing the total treatment time and post-treatment sequelae related to boost administration. In comparison with an external beam boost, IOERT showed a trend towards better local control. Time to in-breast relapse was markedly prolonged, and cosmetic outcome was superior.

## Data Availability

The datasets used and/or analyzed during the current study are available from the corresponding author on reasonable request.

## Abbreviations

BED: Biologically equivalent dose
BT: Brachytherapy
CMF: Cyclophosphamide, methotrexate, fluorouracil
DCIS: Ductal carcinoma in situ
DFS: Disease-free survival
EBRT: External beam radiotherapy
EIC: Extensive intraductal component
EQD2: Equivalent dose in 2-Gy fractions
FU: Follow-up
IBTR: In-breast true recurrence
IOERT: Intraoperative electron radiotherapy
IORT: Intraoperative radiotherapy
LCIS: Lobular carcinoma in situ
LR: Local recurrence
MOSFET: Metal oxide silicon field effect transistors
OS: Overall survival
PTV: Planning target volume
RT: Radiotherapy
SLB: Sentinel node biopsy
WBI: Whole-breast irradiation
