# Correction to: IOERT versus external beam electrons for boost radiotherapy in stage I/II breast cancer: 10-year results of a phase III randomized study

**DOI:** 10.1186/s13058-021-01432-9

**Published:** 2021-04-28

**Authors:** Antonella Ciabattoni, Fabiana Gregucci, Gerd Fastner, Silvio Cavuto, Antonio Spera, Stefano Drago, Ingrid Ziegler, Maria Alessandra Mirri, Rita Consorti, Felix Sedlmayer

**Affiliations:** 1 Department of Radiotherapy, San Filippo Neri Hospital, ASL Roma 1, Rome, Italy; 2 Department of Radiation Oncology, Miulli General Regional Hospital, Acquaviva delle Fonti, Bari, Italy; 3 Department of Radiotherapy and Radio-Oncology, Paracelsus Medical University Hospital Salzburg, Landeskrankenhaus, Salzburg, Austria; 4 Infrastructure Research and Statistics, Clinical Trials and Statistics Unit, AUSL-IRCCS, Reggio Emilia, Italy; 5 Department of Radiotherapy, San Giovanni di Dio Hospital, ASP Agrigento, Agrigento, Italy; 6 Department of Breast and Reconstructive Surgery, Sando Pertini Hospital, Rome, Italy; 7 Medical Physics Unit, San Filippo Neri Hospital, ASL Roma 1, Rome, Italy

**Correction to: Breast Cancer Res 23, 46 (2021)**

**https://doi.org/10.1186/s13058-021-01424-9**

After publication of the original article [1], the authors identified an error in the author name of Ingrid Ziegler.

The incorrect author name is: Ingrid Ziegle.

The correct author name is: Ingrid Ziegler.

The author group has been updated above and the original article [1] has been corrected.
